# Linking servant leadership to followers' thriving at work: self-determination theory perspective

**DOI:** 10.3389/fpsyg.2024.1384110

**Published:** 2024-05-16

**Authors:** Xiaoqun Jiang, Yiyi Wei

**Affiliations:** ^1^School of Management, Guangxi Minzu University, Nanning, China; ^2^School of Labor and Human Resources, Renmin University of China, Beijing, China

**Keywords:** servant leadership, thriving at work, basic psychological need satisfaction, power distance, self-determination theory

## Abstract

Previous studies have confirmed that servant leadership has a positive impact on thriving at work, however, the psychological mechanism in this process has not been fully understood. Based on Self-Determination Theory, this study examines the mediating effect of basic psychological needs and the moderating effect of power distance on the relationship between servant leadership and followers' thriving at work. The results from the between-subject experimental design (Study 1) indicate that servant leadership can satisfy followers' three basic psychological needs. And the results from a questionnaire survey of 455 civil servants at two-time points (Study 2) indicate: (1) Servant leadership has a significantly positive impact on followers' thriving at work; (2) All three basic psychological needs satisfaction serve as a mediator in the relationship between servant leadership and followers' thriving at work; (3) Power distance negatively moderates the relationship between servant leadership and the satisfaction of three basic psychological needs, meaning that the lower on the power distance, the stronger the positive relationship between servant leadership and the satisfaction of three basic psychological needs; (4) Power distance negatively moderates the mediating effects of competence needs satisfaction and relatedness needs satisfaction in the relationship between servant leadership and followers' thriving at work, indicating that the lower on the power distance, the stronger the mediating effects. Our findings highlight the important role of servant leadership in fostering followers' thriving at work and explore the critical role of basic psychological needs satisfaction. This provides empirical evidence to further refine theories regarding thriving at work, and suggests that in order to promote employee thriving, it is important to guide leaders to reevaluating and repositioning their roles.

## 1 Introduction

In today's turbulent economic and business environment, organizations need to rely on thriving employees to maintain competitive advantage and achieve organizational goals (Cao et al., 2022; Goh et al., 2022; Rahaman et al., 2022). Thriving at work is a two-dimensional concept, characterized as the positive psychological state in which individuals jointly experience vitality and learning (Anand et al., 2018). Vitality belongs to the affective dimension, which refers to the feeling that an individual is energetic, enthusiastic and interested in work; Learning belongs to the cognitive dimension, which refers to the sense that an individual is acquiring and applying valuable knowledge and skills (Spreitzer et al., 2005). Thriving at work shares similarities with work engagement, but there are notable distinctions. Both concepts involve energy components, with vitality associated with thriving and vigor with work engagement. However, thriving at work incorporates a distinct element of learning, emphasizing individual growth and development within the workplace. In contrast, work engagement centers on the cognitive and emotional connection between individuals and their work, focusing on absorption in tasks and dedication to job responsibilities (Schaufeli and Bakker, 2010; Niessen et al., 2012). Much has been written about the importance of thriving at work in the academic literature (Walumbwa et al., 2018; Rego et al., 2021). Thriving employees can actively adapt to physical, mental and social adversity with vibrant growth, and they can generate new resources instead of consuming the existing resources (Spreitzer et al., 2012). A meta-analysis found that thriving at work leads to positive employee outcomes (Kleine et al., 2019), including individual health and growth (e.g., physical and mental health, job burnout, career development), work attitude (e.g., organizational commitment, turnover intention) and performance-related outcomes (e.g., work performance, innovation, organizational citizenship behavior). Given its critical role in sustaining individuals and organizations, understanding the antecedents of thriving at work becomes important in fostering sustainable success.

Thriving at work exhibits characteristics of social embeddedness, with vitality and learning deeply embedded in the individual's social systems. Self-development occurs through dynamic interactions with others, particularly interactions with leaders (Spreitzer et al., 2005). Leadership is one of the important antecedents of thriving at work, yet remains understudied. With societal advancements, employees in the workplace not only seek economic rewards but also exhibit pursuits for higher-level mental needs. They prefer leadership styles that are more humane and hope to receive more care, assistance, and support from their leaders (Li and Mao, 2018). Servant leadership is an altruistic leadership style, which has attracted intense attention from scholars and managers in recent years (Eva et al., 2019; Schowalter and Volmer, 2023). Servant leaders do not appear superior to others, they are friendly and prioritize the interests of employees, ultimately achieving organizational goals by fostering followers' growth and development (Liden et al., 2008; van Dierendonck, 2011; Eva et al., 2019; Liao et al., 2021). The meta-analysis by Eva et al. (2019) systematically examined research findings of servant leadership. Specifically, previous studies have explained the influence mechanism of servant leadership mainly from the theoretical perspectives of social exchange theory, social identity theory, social learning theory, resource conservation theory and attribution theory. It is confirmed that servant leadership has significant effects on followers' key work attitudes (such as job engagement, job satisfaction, job meaning and turnover intention, etc.), important organizational behaviors (such as organizational citizenship behavior, innovation behavior, knowledge sharing, helping behavior, initiative behavior, voice behavior, etc.), work performance and wellbeing (Eva et al., 2019).

Although prior studies have evidenced the effectiveness of servant leadership in promoting followers' thriving at work (Walumbwa et al., 2018; Sheikh et al., 2019; Xu and Wang, 2020). However, limited research attention has been paid to the underlying psychological mechanisms through which servant leaders affect followers' thriving at work. According to self-determination theory (SDT), humans have three basic psychological needs: autonomy, competence and relatedness, and individuals tend to develop in a positive direction when these needs are met (Deci and Ryan, 2000). Spreitzer and Porath (2013) further integrated SDT with the concept of thriving at work, proposing an Integrative Model of Human Growth at Work. This model emphasizes the importance of satisfying the three basic psychological needs for individual development and growth, considering them essential nutrients for human flourishing (Spreitzer and Porath, 2013). Servant leadership theory places a greater emphasis on attending to the needs of followers than any other leadership theory (van Dierendonck et al., 2014). Therefore, based on SDT, this study employs the satisfaction of basic psychological needs as the mediating variable to elucidate how servant leadership influences followers' thriving at work by satisfying their three basic psychological needs, respectively.

Beyond the proposed mediating effects, this study also aims to investigate the boundary condition of the relationship, offering further insight into the underlying mechanism. In fact, how followers perceive and interpret leadership behavior is a crucial influencing factor in leadership effectiveness. Previous research on leadership has underpinned the moderating role of power distance in leadership effectiveness (Lian et al., 2012; Anand et al., 2018). Given that power is inherent in hierarchical organizations, and is fundamental to all relationships, employees' perception of power can impact various organizational management processes and outcomes (Daniels and Greguras, 2014). Therefore, we consider power distance as the moderating variable, examining its influence on the effectiveness of servant leadership in fostering thriving at work.

This research contributes to the literature in the following aspects: firstly, based on the social embedding characteristics of thriving at work, we explore the leadership factors that affect followers' thriving at work. This enriches the understanding of the antecedents of thriving at work. Secondly, while prior studies have explored how servant leaders meet the basic psychological needs of their followers, they have not extended to thriving at work (van Dierendonck et al., 2014; Chiniara and Bentein, 2016). Our study bridges this gap by examining the mediating effect of basic psychological needs between servant leadership and thriving at work from the perspective of self-determination theory. Different from other research perspectives (e.g., social exchange theory, social learning theory, attribution theory), SDT captures servant leadership's core tenet of “prioritizing followers' needs” (van Dierendonck et al., 2014). Thus, this study expands the understanding of the psychological mechanism through which servant leadership influences followers' thriving at work. Thirdly, there has been a dearth of experimental research design in the realm of servant leadership studies. In response to recent calls by Eva et al. (2019) and Schowalter and Volmer (2023), we adopted the situational experiment method in study 1 by utilizing a scenario-based experiment design to examine the causal relationship between servant leadership and psychological needs satisfaction. Finally, we identify unique boundary conditions around the relationships between servant leadership and thriving at work. These findings provide valuable managerial insights for promoting workplace thriving.

## 2 Theory and hypotheses

### 2.1 Servant leadership and follower thriving at work

Servant leadership is a holistic leadership style that is “people-centered” but does not ignore performance expectations (Eva et al., 2019; Schowalter and Volmer, 2023). Leaders advocate the belief of “service” and demonstrate personality traits such as modesty, authenticity, and conscientiousness, they care about the needs of their followers, actively share resources, provide guidance, and empower their followers (van Dierendonck, 2011). A recent meta-analysis reveal that servant leadership can lead to several important employee outcomes, including follower attitudes, behaviors, and performance, as well as team and organizational outcomes (Eva et al., 2019). Compared with transformational, authentic, and ethical leadership, servant leadership show greater predicative capability across many outcomes (Eva et al., 2019; Lee et al., 2019; Schowalter and Volmer, 2023). Leaders perceive themselves as service providers, satisfying followers' needs and helping them in their development and success. Thus, servant leaders are increasingly favored by employees (Macedo et al., 2022).

A core assumption of thriving at work is that high levels of both vitality and learning need to be achieved for employees to thrive (Kleine et al., 2019). Scholars posit that while both dimensions of vitality and learning can represent self-growth and personal development in the workplace to some extent, the experience of thriving occurs when these two dimensions mutually reinforce each other (Porath et al., 2012). Thriving at work exhibits social embedding features, and unlike an enduring personality trait, it is a temporary psychological state that can be shaped by the environment (Porath et al., 2012; Van der Walt, 2018). Previous research has pointed out that leadership, leader-follower relationships, and organizational practices are associated with employees' thriving at work (Ren et al., 2022). Thriving at work is conceptualized as a continuum, where individuals experience more or less thriving at any point in time (Spreitzer et al., 2005; Spreitzer and Porath, 2013). As proposed by Deci and Ryan (2000), all individuals have an inherent tendency to pursue growth and development, but the success of this pursuit depends on environmental factors. A meta-analysis has revealed that positive leadership factors, such as supportive leader behaviors, empowerment, and high-quality leader-member exchanges, serve as relational resources for employees to thrive (Kleine et al., 2019). Servant leaders develop followers by prioritizing followers' work needs and interests to achieve organizational sustainability (Jaiswal and Dhar, 2017; Macedo et al., 2022). We posit that servant leaders are humane and wise leaders with a long-term outlook. They focus their efforts on promoting employee thriving to achieve long-term organizational success.

Firstly, servant leaders exhibit traits of altruism and moral reasoning, prioritizing the needs and interests of followers over self-interest. As a result, followers experience an increased sense of growth, empowerment and wellbeing (Lee et al., 2019). When followers feel that their leaders prioritize their growth and wellbeing, they develop higher levels of psychological safety and organizational esteem. This, in turn, fosters their vitality and enhances their work engagement and efficiency (Bao et al., 2018; Walumbwa et al., 2018; Eva et al., 2019). Moreover, servant leaders embrace a “service-first” mindset (Eva et al., 2019), demonstrating care for both the work and lives of followers and providing support. These supportive behaviors help to enhancing the sense of work meaningfulness (Jang et al., 2022), balancing work and family relationships (Tang et al., 2016; Russo et al., 2018; Ren et al., 2022), and alleviating burnout (Tang et al., 2016). Therefore, servant leadership nurtures followers' vitality by meeting their psychological needs of followers.

Secondly, servant leaders create better career development prospects for followers by offering guidance, feedback and work resources to create opportunities for followers to acquire new knowledge, and develop new skills and abilities (Walumbwa et al., 2010). They cultivate a collaborative and participative work environment where leaders and followers jointly tackle difficulties through continuous learning (Sheikh et al., 2019). Furthermore, servant leaders promote empowerment, innovation and future-oriented thinking, meanwhile, encouraging followers to find ways to enhance work performance (Chen et al., 2015; Walumbwa et al., 2018). These management practices contribute to maintaining a continuous learning environment among followers.

Several empirical studies in recent years have confirmed the positive relationship between servant leadership and thriving at work. For example, Jaiswal and Dhar (2017), Walumbwa et al. (2018), and Sheikh et al. (2019) all found that servant leadership is significantly positively correlated with followers' thriving at work. Xu and Wang (2020) found that the developmental and social-emotional support provided by servant leaders helps to establish high-quality team-member exchange relationships, consequently fostering thriving at work among team members. Jang et al. (2022) found that servant leadership promotes thriving at work by enhancing followers' work meaningfulness and workplace spirit. Therefore, we hypothesize that:

*Hypothesis 1: Servant leadership relates positively to followers' thriving at work*.

### 2.2 The mediating role of basic psychological need satisfaction

According to SDT, people have three basic psychological needs: autonomy, competence, and relatedness. These basic psychological needs are the innate, inherent and necessary “lack” of human individuals (Deci and Ryan, 2000). Fundamental to the theory is the principle that various environmental factors can impact the development, growth and health of an individual through the satisfaction of basic psychological needs. Although dispositional differences exist in the strength of these needs, however, SDT asserts that it is the degree to which psychological needs are met, rather than their strength, that determines and shapes personal growth, integrity, and wellbeing (Chiniara and Bentein, 2016). The three basic psychological needs are structurally independent, and the satisfaction of each need can contribute positively to an individual's personal growth and development. Deci et al. (2017) proposed that the influence of various workplace background factors on employee motivation and experience is also mediated by the satisfaction of three basic psychological needs. These basic needs include employees' needs for competence or effectance, relatedness or belongingness and autonomy or self-determination in their work. Servant leadership theory is built on the core assumption that servant leaders focus on meeting followers' needs and establishing a long-term and stable relationship with followers, thereby, influencing organizational outcomes by promoting employees' growth and wellbeing (Liden et al., 2008). Therefore, we need to further explore the relationship between servant leadership and the satisfaction of each psychological need.

Autonomy is considered a prominent need that is fundamental to intrinsic motivation (Chiniara and Bentein, 2016). In the workplace, the satisfaction of employees' autonomy needs mainly comes from two aspects: first, the free will they can experience at work, that is, the feeling of psychological freedom. Second, the freedom to choose how tasks are completed and the ability to work in their own way (Van den Broeck et al., 2016; van Hooff and De Pater, 2019). Servant leadership effectively meets followers' autonomy needs in the following ways: Firstly, servant leaders are forward-thinking managers; they value the intrinsic worth and potential development of followers, respecting their emotions, interests, perspectives, and opinions. They aim to nurture followers' independence and develop a sense of autonomy. This managerial mindset allows the followers to experience more autonomous growth. Secondly, servant leaders advocate power sharing and granting autonomy with the intent of providing followers with opportunities to act independently and make their own decisions (Liden et al., 2008; Newman et al., 2017). When followers perceive their actions as autonomous and self-determined, they experience a heightened sense of autonomy. Thirdly, humility is a fundamental pillar of servant leadership (Sousa and van Dierendonck, 2017; Van Dierendonck et al., 2017). By taking humble positions as servants to followers and respecting them as equal partners rather than exerting command and control, servant leaders can foster mutual trust. This, in turn, creates a reciprocal relationship between leader and followers. Followers are more likely to understand and respect their leaders and experience a greater sense of psychological freedom.

Competence refers to an individual's need to interact effectively with others and have opportunities to use their talents (Deci and Ryan, 2000; Chiniara and Bentein, 2016; van Hooff and De Pater, 2019). At work, employees hope to tackle challenging tasks and achieve desired outcomes. When their competence needs are satisfied, employees experience a sense of accomplishment (Deci et al., 2001). “Helping followers to grow and succeed” is considered one of the important dimensions of servant leadership (Liden et al., 2008). Servant leaders help followers to grow and succeed by actively developing their abilities and providing opportunities for skill improvement or acquiring new ones. They demonstrate genuine concern for followers' career growth and development, they understand followers' interests, abilities, and career goals (Chiniara and Bentein, 2016). Understanding and prioritizing followers' needs enables servant leaders to best match their interests, abilities and goals with their work, enabling them to leverage their abilities and realize their value, and guide them toward optimal career development paths. Moreover, servant leaders offer sufficient autonomy, expressing confidence in followers' ability to excel in their roles. This trust fosters a greater sense of control and competence among followers. A recent study has found a significant positive impact of servant leadership on followers' innovation self-efficacy (Iqbal et al., 2022).

Relatedness refers to the need for connection and maintaining intimacy with others (van Hooff and De Pater, 2019). In organizations, employees seek connection with others, longing to experience care and support, as well as a sense of belonging within their work teams or organization. Servant leaders are relationship-oriented and altruistic-oriented. They demonstrate altruistic sensitivity to the difficulties, concerns, needs and interests of their followers, and interact with them in an open, fair and trustworthy manner. Leaders place particular emphasis on positive emotional communication with followers, offering them emotional support, thereby establishing a long-term, trustworthy, dyadic interaction (Schaubroeck et al., 2011; Özkan et al., 2023). The altruistic mindset of servant leaders, prioritizing followers' interests over self-interests, enhances followers' psychological safety in organizational interpersonal relationships (Jiang and Li, 2020). These supportive behaviors foster a stronger sense of connection for followers with their leader and work teams, and strengthen their sense of belonging within the organization. In addition, servant leaders emphasize a spirit of service that not only to followers, but also to the organization, and other organizational stakeholders (Lemoine et al., 2019). As a result, servant leaders may cultivate positive relationships with followers, who, in turn, reciprocate by engaging in more proactive behaviors.

According to SDT, basic psychological needs satisfaction is the underlying mechanism that motivates and guides people's behavior, and is “necessary for an individual to function effectively and healthily” (Deci and Ryan, 2000). Followers are more likely to experience thriving when their work environment meets their three basic psychological needs (Deci and Ryan, 2008). The relationship between basic psychological needs satisfaction and thriving at work has been empirically supported. Spreitzer and Porath found that the satisfaction of three basic psychological needs, which together explained 54% of the variance of thriving at work, with each need's satisfaction independently predicting thriving at work (Spreitzer and Porath, 2013). Therefore, we hypothesize the following:

*Hypothesis 2a: autonomy need satisfaction mediates the relationship between servant leadership and thriving at work;*

*Hypothesis 2b: competence need satisfaction mediates the relationship between servant leadership and thriving at work;*

*Hypothesis 2c: relatedness need satisfaction mediates the relationship between servant leadership and thriving at work*.

### 2.3 The moderating role of power distance

In social interactions, individuals often assess their own status and power in comparison to others, thereby shaping their perceptions of fairness in power distribution. Under different cultural backgrounds, individuals differ in the extent to which they accept inequality in power. This acceptance of power distribution is termed “power distance” and is considered a cultural value. At the individual level, power distance denotes one's acceptance of inequalities in power within institutions and organizations (Clugston et al., 2000). Recognized as a cornerstone in relationships, power distance can significantly affect many organizational processes and outcomes (Daniels and Greguras, 2014; Anand et al., 2018). Individuals higher on power distance perceive distinctions based on power or hierarchical positions, and believe that organizational authority should be respected, showing loyalty and obedience to leaders. Whereas individuals lower on power distance view leaders and followers as having equal status, and that everyone in the organization should have the right to express opinions and participate in decision-making (Daniels and Greguras, 2014).

From the perspective of leader-follower fit, leadership effectiveness may be enhanced when the leadership style aligns with the followers' power distance orientation. Servant leadership is a “bottom-up” managerial approach wherein leaders respect followers' advice and opinions, and help followers to grow and succeed. Most importantly, servant leaders do not show superiority and place the satisfaction of followers' needs over their own. These leader behaviors may deviate from the expectations and perceptions that followers higher on power distance have for their leaders. They may feel that is not a true trait of a leader, so they may act more cautiously in their interactions with servant leaders and try to maintain a more prudent distance from them. In this sense, for followers higher on power distance, the effect of servant leadership on the satisfaction of followers' psychological needs may be diminished. In contrast, followers lower on power distance are more willing to establish a close relationship with their leader, expecting support in career development and work-family balance from leaders. The traits and behaviors of servant leaders are thus more likely to satisfy their basic psychological needs. Therefore, we hypothesize that:

*Hypothesis 3a: Power distance negatively moderates the relationship between servant leadership and autonomy need satisfaction;*

*Hypothesis 3b: Power distance negatively moderates the relationship between servant leadership and competence need satisfaction;*

*Hypothesis 3c: Power distance negatively moderates the relationship between servant leadership and relatedness need satisfaction*.

Combining Hypothesis 2 and Hypothesis 3, this study further anticipates that power distance will negatively moderate the mediating role of basic psychological needs. That is, the mediating effect of basic psychological needs satisfaction in the relationship between servant leadership and thriving at work is expected to be moderated by power distance. A lower power distance is expected to strengthen the positive relationship between servant leadership and thriving at work through the mediating role of basic psychological needs satisfaction. Conversely, a higher power distance is expected to weaken the relationship through the mediating role. We therefore propose the following hypotheses:

*Hypothesis 4a: Power distance negatively moderates the mediating effect of autonomy need satisfaction;*

*Hypothesis 4b: Power distance negatively moderates the mediating effect of competence need satisfaction;*

*Hypothesis 4c: Power distance negatively moderates the mediating effect of relatedness need satisfaction*.

**Figure 1:**
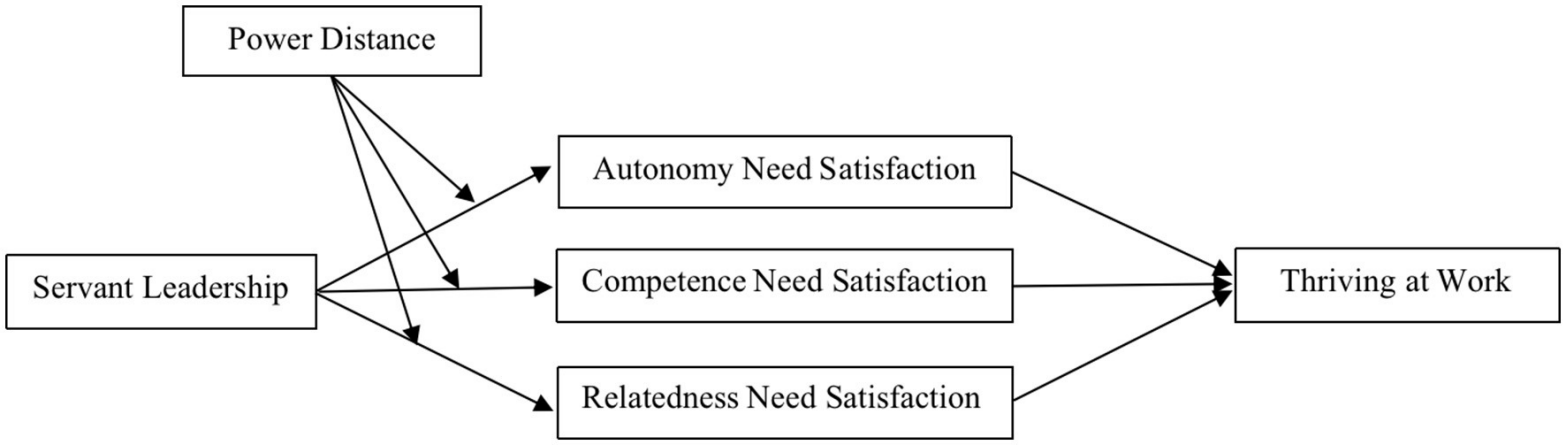
Hypothesized model.

All hypotheses were combined into one comprehensive research model (Figure 1).

## 3 Study 1

### 3.1 Participants and design

Sixty-six students enrolled in the MBA course at a business school in China's university voluntarily participated in the study. The sample comprised 30 males and 36 females, with a mean age of 32.29 years (SD = 4.26). The participants were randomly assigned to one of two experimental scenarios using a between-subject design: (1) high servant leadership; (2) low servant leadership (n= 33 for each group). Then, participants provided demographic information, read the material, and were instructed to imagine their supervisor as described in the material. Following this, participants answered questions about how well their basic psychological needs would be satisfied while working with their immediate supervisor.

### 3.2 Experimental materials

We developed the materials based on the behavioral description of servant leadership by Eva et al. (2019) and the relevant script developed by van Dierendonck et al. (2014).

High servant leadership is described as follows:

*He/she has often assisted you with your work and life*.

*He/she is a person of humility, integrity, honesty and sincerity, and shares his/her thoughts and feelings with you*.

*He/she constantly listened to your opinions, and did not take one employees' side over another*.

*He/she tolerates mistakes, and provides freedom so you can develop your own abilities*.

*He/she values ethical standards and emphasizes that it is more important to do the right thing than looking good in front of your workmates*.

*He/she shows great humanity, and understanding of your personal needs and standpoint*.

Low servant leadership is described as follows:

*He/she rarely assists you with your work and life*.

*He/she is proud, hypocritical and rarely shares his/her thoughts and feelings*.

*He/she rarely listens to you and sometimes favor one employees' side over another.....*.

*He/she is not allowed to make mistakes and must do the job according to his/her requirements and manner*.

*He/she emphasizes how to achieve a set goal, regardless of whether it violates ethical standards*.

*He/she is not very human, does not care about your personal needs, and does not understand your position*.

### 3.3 Measures

After reading the material, participants completed a survey. Basic psychological needs satisfaction was measured using the La Guardia et al. (2000) scale. The scale contains three dimensions: autonomy satisfaction, competence satisfaction and relatedness satisfaction, with each dimension comprising three items. Sample items include “When I am with my supervisor, I feel free to be who I am”, “When I am with my supervisor, I feel like a competent person” and “When I am with my supervisor, I feel closeness and intimacy”. The Cronbach's α of the total scale is 0.924, and the Cronbach's α of the three dimensions is 0.861, 0.742, and 0.813 respectively. All items were measured on a 7-point Likert scale (1 = strongly disagree, 7 = strongly agree).

### 3.4 Results

We conducted a one-way analysis of variance (ANOVA), and the results showed that the main effect of servant leadership on the satisfaction of the three basic psychological needs was significant. The satisfaction of followers' autonomy needs [F_(1, 74)_ = 203.310, *p* < 0.001, η^2^ = 0.733] in high servant leadership condition (M = 16.553, SD = 2.638) are significantly higher than that under low servant leadership condition (M = 7.42, SD = 2.937);competence needs [F_(1, 74)_ = 47.911, *p* < 0.001, η^2^ = 0.393] in high servant leadership condition (M = 16.316 SD = 2.682) are significantly higher than that under low servant leadership condition (M = 10.711, SD = 4.210); relatedness needs [F_(1, 74)_ = 91.315, *p* < 0.001, η^2^ = 0.552] in high servant leadership condition (M = 12.078, SD = 2.148) are significantly higher than that under low servant leadership condition (M = 7.395, SD=2.125). Hence, Hypothesis 2a, Hypothesis 2b and Hypothesis 3c are supported.

In sum, the results of Study 1 show that servant leadership is positively related to the satisfaction of followers' basic psychological needs. In order to further examine the impact of basic psychological need satisfaction on thriving at work and the mediating role of basic psychological need satisfaction between servant leadership and thriving at work, we conducted study 2. To improve the external validity, Study 2 will reexamine the conclusions of Study 1 through a questionnaire survey, and further examine other hypotheses.

## 4 Study 2

### 4.1 Method

#### 4.1.1 Sample and procedure

The researchers contacted 50 civil servants working in government institutions across 25 provinces and municipalities in China (e.g., Beijing, Shandong, Hebei, Guangxi, and Yunnan), and asked them to help collect data in their organization.

To reduce common method bias, we collected data from paper-based and web-based surveys simultaneously at two-time points. At Time 1, a total of 634 civil servants took part in the survey and rated on the Servant Leadership and Basic Psychological Needs Satisfaction Scale. One month later, the same participants rated on Power Distance and Thriving at Work Scales. We matched the data from the two surveys and screened out the ineligible data, resulting in 455 valid records at two-time points, and the effective response rate was 71.77%.

To ensure the data from the two surveys can be accurately matched, the contacts were required to make a record of questionnaire distribution at the first round. In specific, the paper-based survey was matched according to the questionnaire number, and the contacts distributed surveys centrally and collected them on the spot. The web-based survey was completed through a mobile social networking platform (i.e., Wechat). The contacts forwarded the questionnaire link to the participants via WeChat and matched their questionnaire according to their WeChat ID number.

Of the total sample respondents, 54.7% were male and 45.3% were female. With regard to age, 33.6% were under 30, 45.8% were between 31 and 40, 18.0% were between 41 and 50, and 2.6% were over 50. In terms of administrative rank, clerks account for 44.4%, deputy section chiefs 19.1%, section chiefs 24.0%, deputy department heads 7.0%, and department heads 5.5%. In terms of academic qualifications, 5.1% were junior college-educated or lower educated, 61.5% hold bachelor's degrees, 30.7% hold master's degrees and 2.7% hold doctoral degrees.

#### 4.1.2 Measures

##### 4.1.2.1 Servant leadership

Servant leadership was measured using the scale developed by Liden et al. (2015). This scale is the shortened version of the Servant Leadership Scale developed by Liden et al. (2008). The scale consists of seven items, a sample item includes “My leader puts my best interests ahead of his/her own”. Each item was rated on a 7-point Likert scale, ranging from 1 (strongly disagree) to 7 (strongly agree). In this study, Cronbach's α of this scale was 0.870.

##### 4.1.2.2 Basic psychological needs satisfaction

Basic psychological needs satisfaction was measured using the Work-related Basic Need Satisfaction Scale (W-BNS) developed by Van den Broeck et al. (2010). The scale consists of three different sub-scales: autonomy satisfaction, competence satisfaction and relatedness satisfaction, each of which contains 6 items. We remove one item whose CITC value is < 0.5 on the autonomy satisfaction scale, leaving five items. A sample item for autonomy satisfaction includes “I feel free to do my job the way I think it could best be done”. We have reserved 6 items of the competence satisfaction scale, and its sample item includes “I feel competent at my job”. We removed one item with a CITC value below 0.5 on the relatedness satisfaction scale, leaving five items. A sample item for relatedness satisfaction includes “Some people I work with are close friends of mine”. Each item was rated on a 5-point Likert scale, ranging from 1 (strongly disagree) to 5 (strongly agree). In this study, Cronbach's α were 0.818, 0.853, and 0.785 respectively.

##### 4.1.2.3 Power distance

The scale used to capture the followers' perceptions of power was obtained from Dorfman and Howell (1988). The scale consists of five items, a sample item includes “Leaders should make most decisions without consulting followers”. Each item was rated on a 5-point Likert scale, ranging from 1 (strongly disagree) to 5 (strongly agree). In this study, Cronbach's α of this scale was 0.755.

##### 4.1.2.4 Thriving at work

Participants reported their thriving at work using the scale developed by Porath et al. (2012). This scale consists of ten items, five of which assess the individuals' state of learning (e.g., “I find myself learning often”), while the other five items assess the individuals' vitality (e.g., “I feel alive and vital”). Each item was rated on a 5-point Likert scale, ranging from 1 (strongly disagree) to 5 (strongly agree). In this study, Cronbach's α of this scale was 0.907.

##### 4.1.2.5 Control variables

Previous studies have found that age, position and educational background are related to thriving at work to a certain extent (Kleine et al., 2019). In addition, women are more likely to feel tired and less energetic than men (Niessen et al., 2012; Jiang et al., 2019). Therefore, demographic variables such as gender, age, administrative level and education were used as control variables in this study.

### 4.2 Results

This study conducted statistical analyses on the data using SPSS 24.0 and Mplus 8.0. First, Mplus 8.0 was adopted for confirmatory factor analysis. Second, descriptive statistics and correlation analysis were conducted using SPSS 24.0. Third, hypothesis testing was performed using the PROCESS in SPSS.

#### 4.2.1 Confirmatory factor analyses

We used Mplus 8.0 to conduct confirmatory factor analysis to examine the discriminant validity of the study variables (servant leadership, autonomy need satisfaction, competence need satisfaction, relatedness need satisfaction, power distance, and thriving at work). The results are shown in Table 1. The fitting index of the six-factor model is the best compared to other models (χ^2^*/df* = 2.360, CFI = 0.907, TLI = 0.897, RMSEA = 0.055, SRMR = 0.059). The fitting index for the four-factor, three-factor, two-factor, and single-factor models are not ideal, and each of them decreases as the number of factors decreases. Therefore, the results confirm the good discriminant validity of our study variables.

**Table 1:** The result of confirmatory factor analyses.

**Model**	***χ^2^/df***	***CFI***	***TLI***	***RMSEA***	***SRMR***
Six-factor model (*SL, AN, CN, RN, TH, PD*)	2.360	0.907	0.897	0.055	0.059
Four-factor model (*SL, AN* + *CN* + *RN, TH, PD*)	3.284	0.842	0.826	0.071	0.075
Three-factor Model (*SL, AN* + *CN* + *RN* + *TH, PD*)	3.427	0.831	0.815	0.073	0.078
Two-factor model (*SL* + *AN* + *CN* + *RN* + *TH, PD*)	4.156	0.780	0.760	0.083	0.087
One-factor model (*SL* + *AN* + *CN* + *RN* + *TH* + *PD*)	4.302	0.770	0.749	0.085	0.087

n = 455; χ^2^ = chi-square statistic; CFI, comparative fit index; TLI, Tucker-Lewis index; RMSEA, root mean square error of approximation; SRMR, standardized root mean square residual; SL, servant leadership; AN, autonomy need satisfaction; CN, competence need satisfaction; RN, relatedness need satisfaction; TH, thriving at work; PD, power distance.

#### 4.2.2 Common method bias

To minimize the impact of common method bias, we collected data at two-time points and exercised strict procedural controls during the investigation. We used Harman's single-factor test to assess common method bias. The results showed that the highest single factor contributed was 31%, less than the 40% cut-off value, suggesting no CMV in the data. Moreover, we also employed the common latent factor technique by Podsakoff et al. (2003). The results show that when a method factor is added to the six-factor model (χ^2^*/df* = 2.505, CFI = 0.896, TLI = 0.886, RMSEA = 0.058, SRMR = 0.067), the model fitting index decreases (ΔCFI = −0.011, ΔTLI = −0.011, ΔRMSEA = 0.003, ΔSRMR = 0.008). This further indicates that the common method bias is no threat to this study.

### 4.3 Descriptive statistics

The mean, standard deviation and correlation of the study variables are presented in Table 2. Servant leadership was significantly positively correlated with autonomy need satisfaction, competence need satisfaction, relatedness need satisfaction, and thriving at work (*r* = 0.593, *p* < 0.001; *r* = 0.248, *p* < 0.001; *r* = 0.527, *p* < 0.001; *r* = 0.593, *p* < 0.001; *r* = 0.494, *p* < 0.001), autonomy need satisfaction, competence need satisfaction, and relatedness need satisfaction were significantly positively correlated with thriving at work (*r* = 0.549, *p* < 0.001; *r* = 0.592, *p* < 0.001; *r* = 0.577, *p* < 0.001), power distance was significantly negatively correlated with servant leadership, autonomy need satisfaction, competence need satisfaction, relatedness need satisfaction and thriving at work (*r* = −0.323, *p* < 0.001; *r* = 0.298, *p* < 0.001; *r* = −0.271, *p* < 0.001; *r* = −0.393, *p* < 0.001; *r* = −0.327, *p* < 0.001).

**Table 2:** Means, standard deviations, and correlations among the variables.

	**M**	**SD**	**1**	**2**	**3**	**4**	**5**	**6**	**7**	**8**	**9**
1. Servant leadership	4.600	1.316	——								
2. Autonomy need satisfaction	2.924	0.863	0.593^***^	——							
3. Competence need satisfaction	3.886	0.724	0.248^***^	0.426^***^	——						
4. Relatedness need satisfaction	3.760	0.816	0.527^***^	0.534^***^	0.488^***^	——					
5. Power distance	2.383	0.736	−0.323^***^	−0.298^***^	−0.271^***^	−0.393^***^	——				
6. Thriving at work	3.741	0.782	0.494^***^	0.549^***^	0.592^***^	0.577^***^	−0.327^***^	——			
7. Gender	1.453	0.498	−0.182^**^	−0.084	−0.159^**^	−0.150^**^	0.039	−0.221^***^	——		
8. Age	1.897	0.783	0.008	0.041	0.182^**^	0.112^*^	−0.015	0.160^**^	−0.207^***^	——	
9. Administrative rank	2.101	1.205	0.154^**^	0.105^*^	0.211^***^	0.203^***^	−0.149^**^	0.237^***^	−0.293^***^	0.520^***^	——
10. Educational	2.385	1.576	0.106^*^	0.032	0.010	0.056	−0.049	0.080	0.036	−0.105^*^	0.242^***^

n = 455; M, mean; SD, standard deviation; ^*^p < 0.05, ^**^p < 0.01, ^***^p < 0.001.

### 4.4 Results of proposed hypotheses

We used the PROCESS in SPSS 24.0 and selected Model 4 to test the mediating effect with servant leadership as the independent variable, thriving at work as the dependent variables, autonomy, competence and relatedness need satisfaction as the mediating variables, while incorporating gender, age, administrative level and education as control variables. As shown in Model 4 in Table 3, servant leadership has a significant positive predictive influence on thriving at work (β = 0.461, *p* < 0.001). Hypothesis 1 is supported. When servant leadership and the satisfaction of the three basic psychological needs simultaneously predict thriving at work (Model 5 in Table 3), the satisfaction of the three basic psychological needs has a significant positive influence on thriving at work (β = 0.179, *p* < 0.001; β = 0.353, *p* < 0.001; β = 0.193, *p* < 0.001), while the direct effect of servant leadership is still significant (β = 0.176, *p* < 0.001). The findings suggest that the satisfaction of three basic psychological needs partially mediates the relationship between servant leadership and thriving at work. Hypothesis 2 is supported.

**Table 3:** Hierarchical regression: the mediating effect of servant leadership on thriving at work.

	**Autonomy need satisfaction**		**Competence need satisfaction**		**Relatedness need satisfaction**		**Thriving at work**		**Thriving at work**	
	**Model 1**		**Model 2**		**Model 3**		**Model 4**		**Model 5**	
	β	**SE**	β	**SE**	β	**SE**	β	**SE**	β	**SE**
Gender	0.076	0.081	−0.127	0.096	−0.035	0.085	−0.187^*^	0.085	−0.149^*^	0.069
Age	0.042	0.059	0.137^*^	0.070	0.067	0.062	0.123^***^	0.062	0.054	0.051
Administrative rank	0.011	0.041	0.090	0.048	0.079	0.043	0.069	0.043	0.020	0.035
Education	−0.021	0.026	−0.016	0.031	−0.009	0.027	0.016	0.027	0.027	0.022
Servant leadership	0.601^***^	0.039	0.221^***^	0.046	0.510^***^	0.041	0.461^***^	0.041	0.176^***^	0.043
Autonomy need satisfaction									0.179^***^	0.044
Competence need satisfaction									0.353^***^	0.039
Relatedness need satisfaction									0.193^***^	0.043
F	49.403^***^		10.782^***^		37.729^***^		35.810^***^		64.984^***^	
R^2^	0.355		0.107		0.296		0.285		0.538	

n = 455, ^*^p < 0.05, ^***^p < 0.001.

To further clarify the mediating effect, we performed a Bootstrap test. Data analysis results show that the total effect of servant leadership on thriving at work is 0.461, with a 95% confidence interval of [0.380, 0.541]. Table 4 shows the results of direct effect, indirect effect, and difference comparison. The mediation effect index of autonomy need satisfaction is 0.108, the 95% confidence interval is [0.050, 0.166], excluding 0; the mediation effect index of competence need satisfaction is 0.078, and the 95% confidence interval is [0.039, 0.125], excluding 0; the mediating effect index of relatedness need satisfaction is 0.099, and the 95% confidence interval is [0.054, 0.148], excluding 0. The findings suggest that the mediating effect of satisfaction of the three basic psychological needs is significant. The direct effect accounted for 38.18% of the total effect. The mediating effects of autonomy, competence and relatedness needs satisfaction accounted for 23.43%, 16.91% and 21.48% of the total effect respectively. Moreover, we compared the differences in the mediating effects of the satisfaction of the three psychological needs. The findings show that the confidence intervals of the difference coefficients all contain 0, indicating that there is no significant difference between the mediating effect indexes of the three psychological needs satisfaction.

**Table 4:** Decomposition of mediation effects.

	**Type of effect**	**Effect**	**SE**	**95% LLCI**	**95% ULCI**	**%**
Direct effect		0.176	0.043	0.091	0.261	38.18%
Indirect effects	Autonomy need satisfaction	0.108	0.029	0.050	0.166	23.43%
	Competence need satisfaction	0.078	0.022	0.039	0.125	16.91%
	Relatedness need satisfaction	0.099	0.024	0.054	0.148	21.48%
Difference comparison	Autonomy need satisfaction-Competence need satisfaction	0.030	0.038	−0.046	0.102	
	Autonomy need satisfaction-Relatedness need satisfaction	0.009	0.039	−0.070	0.085	
	Competence need satisfaction-Relatedness need satisfaction	−0.020	0.032	−0.080	0.043	

### 4.5 Moderated mediation effects

We used Model 7 of the PROCESS program to test moderating effects and moderated mediating effects. Controlling for gender, age, administrative level and education, we used servant leadership as the independent variable, power distance as the moderating variable, autonomy, competence and relatedness need satisfaction as the mediating variables, and thriving at work as the dependent variables to test the moderated mediating effects of the first half of the model path.

**Figure 2:**
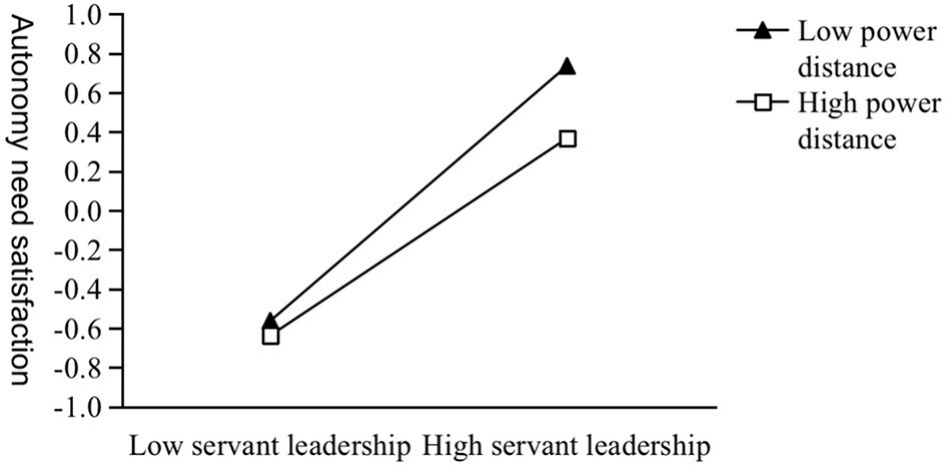
The moderating effect of power distance on servant leadership and autonomous need satisfaction.

**Figure 3:**
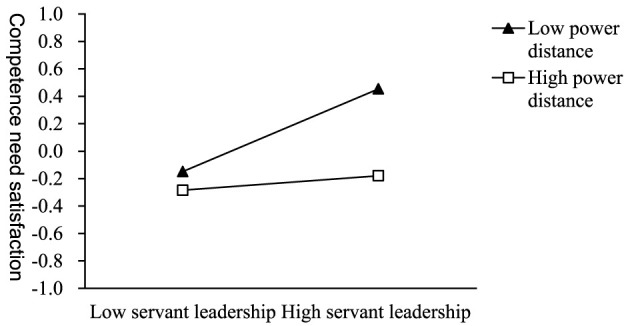
The moderating effect of power distance on servant leadership and competence need satisfaction.

**Figure 4:**
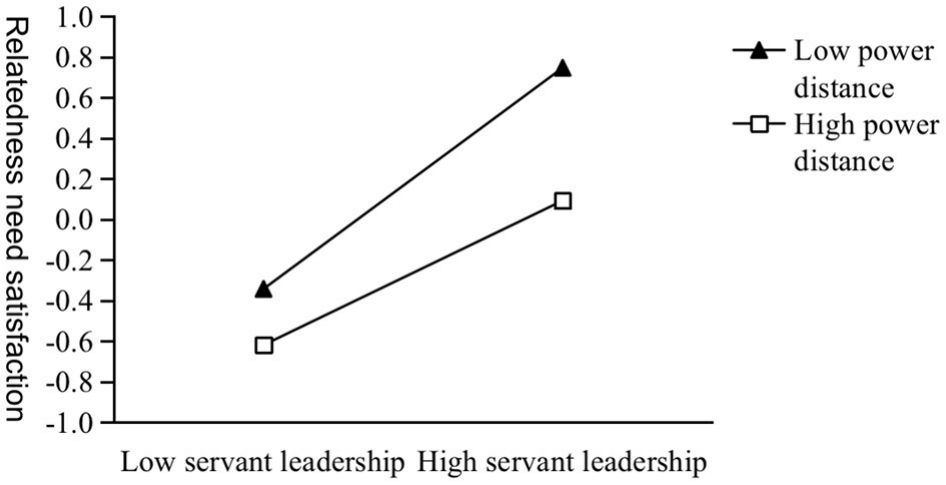
The moderating effect of power distance on servant leadership and relatedness need satisfaction.

As Model 1–3 shown in Table 5, the interaction term of servant leadership and power distance has a significant negative impact on the satisfaction of autonomy needs, competence needs and relatedness needs (β = −0.074, *p* < 0.001; β = −0.124, *p* < 0.001; β = −0.095, *p* < 0.001). This result indicates that H3a, H3b, and H3c are supported. We conducted simple slope analyses and plotted three simple slope graphs (Figures 2–4). For followers lower on power distance, servant leadership significantly predicted their autonomy, competence, and relatedness needs satisfaction (β = 0.649, *p* < 0.001; β = 0.301, *p* < 0.001; β = 0.544, *p* < 0.001). For followers higher on power distance, servant leadership can also significantly predict their autonomy and relatedness needs satisfaction (β = 0.502, *p* < 0.001; β = 0.355, *p* < 0.001), except for competence needs satisfaction (β = 0.053, *p* > 0.05).

**Table 5:** Hierarchical regression: the moderation effects of power distance.

**Variables and statistic**	**Autonomy need satisfaction**		**Competence need satisfaction**		**Relatedness need satisfaction**	
	**Model 1**		**Model 2**		**Model 3**	
	β	**SE**	β	**SE**	β	**SE**
Gender	0.059	0.080	−0.158	0.093	−0.067	0.081
Age	0.060	0.059	0.167	0.068	0.096	0.060
Administrative rank	−0.015	0.041	0.045	0.047	0.033	0.042
Education	−0.016	0.026	−0.008	0.030	−0.002	0.026
Servant leadership	0.576^***^	0.041	0.177^***^	0.047	0.450^***^	0.042
Power distance	−0.111^**^	0.040	−0.192^***^	0.046	−0.233^***^	0.041
Servant leadership × power distance	−0.074^*^	0.037	−0.124^**^	0.042	−0.095^*^	0.037
*F*	37.981^***^		12.221^***^		35.470^***^	
*R^2^*	0.373		0.161		0.357	

n = 455, ^*^p < 0.05, ^**^p < 0.01, ^***^p < 0.001.

Then, in order to examine the moderating effect of power distance on the mediating effect of the satisfaction of three basic psychological needs respectively, we conducted a conditional mediating effects analysis. Results are shown in Table 6. In the path of Servant Leadership → Satisfaction of Autonomy Needs → Thriving at work, the moderated mediation effect value is −0.013, with a 95% confidence interval of [-0.031, 0.000], including 0. This indicates that the moderated mediation effect in this path is not significant. Therefore, Hypothesis 4a is not supported.

**Table 6:** Results for conditional indirect effect analysis.

**Index of moderated mediation**		**Effect**	**SE**	**95% LLCI**	**95% ULCI**
SL → AN → TH		−0.013	0.008	−0.031	0.000
Power distance	M – SD	0.116	0.032	0.055	0.181
	M + SD	0.090	0.027	0.039	0.145
	Constrast	−0.026	0.016	−0.062	0.001
SL → CN → TH		−0.044	0.019	−0.083	−0.009
Power distance	M – SD	0.106	0.029	0.056	0.162
	M + SD	0.019	0.028	−0.037	0.075
	Constrast	−0.088	0.038	−0.167	−0.017
SL → RN → TH		−0.018	0.009	−0.038	−0.002
Power distance	M – SD	0.105	0.026	0.056	0.160
	M + SD	0.069	0.020	0.034	0.111
	Constrast	−0.037	0.018	−0.076	−0.005

Bootstrap size = 5,000, SL, servant leadership; AN, autonomy need satisfaction; CN, competence need satisfaction; RN, relatedness need satisfaction; TH, thriving at work; PD, power distance.

In the path of Servant Leadership → Satisfaction of Competence Needs → Thriving at work, the moderated mediation effect value is −0.044, with a 95% confidence interval of [−0.083, −0.009], excluding 0. This indicates that the mediating effect of competence needs satisfaction is negatively moderated by power distance. For followers lower on power distance, competence needs satisfaction mediates the relationship between servant leadership on thriving at work (mediation effect value: 0.106, 95% confidence interval: [0.056, 0.162]). In contrast, for followers higher on power distance, the mediated effect of servant leadership through competence needs satisfaction on thriving at work is not significant (mediation effect value: 0.019, 95% confidence interval: [−0.037, 0.075]). Additionally, the group difference comparison results show a significant difference between the two, with a difference of −0.088 and a 95% confidence interval of [−0.167, −0.017], not including 0. Therefore, Hypothesis 4b is supported.

In the path of Servant Leadership → Satisfaction of Relatedness Needs → Thriving at work, the moderated mediation effect value is −0.018, with a 95% confidence interval of [−0.038, −0.002], excluding 0. This indicates that the mediating effect of relatedness needs satisfaction between servant leadership and thriving at work is negatively moderated by power distance. For followers lower on power distance, relatedness needs satisfaction mediates the relationship between servant leadership on thriving at work (mediation effect values of 0.105, 95% confidence interval: [0.056, 0.160]). Similarly, for followers higher on power distance, relatedness needs satisfaction mediates the relationship between servant leadership on thriving at work (mediation effect values of 0.069, 95% confidence interval: [0.034, 0.111]). Moreover, the group difference comparison results show a significant difference between the two, with a difference of −0.037 and a 95% confidence interval of [−0.076, −0.005], excluding 0. Therefore, Hypothesis 4c is supported.

## 5 Discussion

While prior research has discussed the various impact of leadership styles on thriving at work (Niessen et al., 2017; Xu and Wang, 2020), limited research attention has been given to servant leadership. Our findings highlight the important role of servant leadership in fostering followers' thriving at work, consistent with previous studies by Chen et al. (2015) and Sheikh et al. (2019). Similarly, our findings also support the viewpoint of scholars like Spreitzer et al. (2005), suggesting that thriving at work requires more than just eliminating or reducing stressors; instead, it necessitates the introduction of favorable contextual factors in the workplace. Servant leadership is a type of leadership that is committed to achieving organizational thriving through employee thriving. Leaders constantly meet the various needs of their followers, helping them grow and develop new knowledge toward organizational goals (Macedo et al., 2022), and organizations can sustain long-term thriving.

We provide empirical evidence that servant leadership influences followers' thriving at work through a multiple mediation pathway, including the satisfaction of autonomy, competence, and relatedness needs. The social embeddedness model and the self-growth integration model, grounded in self-determination theory, posit that one important psychological mechanism between leadership behavior and thriving at work is the satisfaction of basic psychological needs (Spreitzer et al., 2005; Spreitzer and Porath, 2013). Employees are more likely to thrive when the work environment facilitates the satisfaction of employees' autonomy, competence, and relatedness needs (Deci and Ryan, 2008). In managerial practices, leaders often prioritize followers' contributions and value to the organization, overlooking the significance of psychological needs satisfaction and the ways to meet these needs. This study empirically supported the mediating role of the three basic psychological needs satisfaction in the relationship between servant leadership and followers' thriving at work, and highlighting servant leadership's contribution to meeting these needs. The comparative analysis further reveals no significant differences in the mediating effects of the three psychological needs satisfaction, highlighting their equally crucial roles in the process through which servant leadership influences followers' thriving at work.

Values shape the reactions of followers to leader behavior, because their values affect the ways in which followers perceive their leaders. Power distance, as an individual-level value, is one crucial determinant of leadership effectiveness (Kirkman et al., 2009; Anand et al., 2018). Particularly in countries higher on power distance, such as China (Dorfman and Howell, 1988), show high respect for hierarchy and formal authority. Followers' perceptions of power and status are likely to influence the effectiveness of servant leadership in promoting thriving at work. This study found that power distance played a negative moderating role in the relationships between servant leadership and the satisfaction of three basic psychological needs, indicating a mismatch between followers' power distance and the values manifested by servant leadership. Those higher on power distance, tend to accept power inequality, respecting the authority and relying on their directives (Daniels and Greguras, 2014; Anand et al., 2018). However, servant leaders, exhibiting characteristics and traits contrary to what followers expect from a true leader, may be perceived as less sincere. As a result, servant leadership effectiveness in meeting their basic psychological needs is greatly diminished. In contrast, followers low on power distance find the management philosophy of servant leadership is more aligned with their own standpoints (Daniels and Greguras, 2014; Anand et al., 2018), making servant leadership more effective in meeting their basic psychological needs. This outcome provides additional insights into the boundary conditions surrounding the mediating role of basic psychological need satisfaction.

In addition, the mediating effects (i.e., competence needs satisfaction and relatedness needs satisfaction) are also moderated by power distance. That the indirect effects of servant leadership on followers' thriving at work were stronger when followers lower on power distance. This finding provides further clarification on the boundary conditions under which servant leadership can better promote the thriving of the followers through the mediating role of basic psychological needs satisfaction.

### 5.1 Theoretical implications

This paper has three main contributions:

Firstly, this study identifies servant leadership as a predictor of followers' thriving at work, thereby enhancing our understanding of the antecedents of thriving at work. Prior studies have initially explored the influence of several types of leadership factors such as authentic leadership (Mortier et al., 2016), transformational leadership (Niessen et al., 2017; Hildenbrand et al., 2018), servant leadership (Sheikh et al., 2019; Xu and Wang, 2020; Jang et al., 2022), family supportive superiors (Russo et al., 2018), leadership helping behaviors (Chen et al., 2020), and gritty Leaders (Rego et al., 2021) on thriving at work. However, the wide array of leadership variables and the limited number of studies to date have hindered the understanding of the genuine relationship between specific leadership factors and thriving at work. Our research finds that servant leadership can promote followers' thriving at work, which enriches our understanding of the social embeddedness of thriving at work and its antecedents.

Secondly, while two previous studies explored the impact of servant leadership on thriving at work from the perspectives of social exchange theory, social learning theory, conservation of resource theory and socially embedded model of thriving (Sheikh et al., 2019; Xu and Wang, 2020; Jang et al., 2022), the underlying psychological mechanism through which servant leadership promotes thriving at work has not been deeply explored. We respond to the call from scholars like Eva et al. (2019) to deepen our understanding of how servant leadership influences followers' thriving. Drawing on SDT, this study empirically examined the mediating role of three basic psychological needs satisfaction in the relationship between servant leadership and thriving at work. According to SDT, the degree of self-determination is reflected in the extent of basic needs satisfaction, directly influencing the internalization of both intrinsic and extrinsic motivation, thereby promoting individual thriving. In addition, the three basic psychological needs are structurally independent and can individually predict outcome variables (Van den Broeck et al., 2016). In this sense, the study explored the separate mediating effects of the satisfaction of three basic needs, suggesting no difference in their impacts. This provides empirical evidence to further refine theories regarding thriving at work.

Thirdly, our research provides additional insights into the boundary conditions that influence how leadership impacts followers' thriving at work. While most scholars have focused on perceived interpersonal justice, perceived leader support, political climate, and other environmental perception variables as boundary conditions for predicting thriving at work (Xu and Wang, 2020; Rego et al., 2021; Jang et al., 2022), we take power distance, one of the cultural values, as the moderating variable, and find that power distance negatively moderates the mediating effect of the three basic needs satisfaction between servant leadership and thriving at work. Therefore, our study contributes to the literature on thriving at work by highlighting power distance as a significant boundary condition. Additionally, we expand the research field of the interaction between power distance and leadership characteristics (Lian et al., 2012).

### 5.2 Practical implications

The study also has several practical implications:

(1) Employee thriving is not only about individual health, growth, and career development but also a crucial factor influencing organizational performance. In promoting employee thriving, it is important to guide leaders to reevaluating and repositioning their roles. Leaders who focus on serving the needs of followers may foster their vitality and learning. Organizations can adopt the servant leadership approach and integrate the concept of “service” into leader selection, training, evaluation, and organizational culture.
(2) In managerial practice, leaders often emphasize followers' contributions to the organization, while neglecting the importance of meeting their psychological needs. This study found positive association between servant leadership and followers' thriving through the mediating role of basic psychological needs satisfaction. In specific, leaders should meet followers' autonomy needs by empowering them, fostering their own decision-making capabilities. Secondly, leaders should meet followers' competence needs by providing opportunities for learning and growth, helping them enhance their job skills, and making them experience sense of learning and growing. Thirdly, leaders should address followers' relatedness needs by establishing long-term, mutually trusting relationships, caring about their needs and interests, and strengthening followers' sense of belonging to the organization.
(3) Due to the impact of followers' power distance on leadership effectiveness, leaders can adopt differentiated managerial practices. For followers higher on power distance, leaders can minimize consulting their opinions, meanwhile, simply giving assignments and instructions. For followers lower on power distance, leaders can engage in more communication with them, and provide them with more opportunities in decision-makings.

### 5.3 Limitations and future directions

The study has several limitations that indicate future research avenues. Firstly, the scenario experiment of Study 1 describes two leadership styles: high servant leadership and low servant leadership. These hypothetical scenarios leads to non-consequential outcomes and limited causal inferences (Schowalter and Volmer, 2023). Future research could design field experiments or laboratory experiments to examine the effects of servant leadership. Secondly, the data of all variables in this study are self-reported. Although common method bias analyses show no threat to this study, future research should consider multi-source data. Thirdly, this study uses followers' perception of servant leadership to estimate the impact of servant leadership on thriving at work. However, Schowalter and Volmer (2023) recently pointed out that this measurement may pose a threat to casual inferences. Future studies may investigate alternative methods such as situational judgment tests to draw a more scientific implication. Finally, this study only discussed the relationship between servant leadership and thriving at work from the individual level. We might consider using a cross-level research design to investigate the impact of servant leadership on thriving at work at the team and organizational levels.

## Data availability statement

The raw data supporting the conclusions of this article will be made available by the authors, without undue reservation.

## Ethics statement

Ethical review and approval was not required for the study on human participants in accordance with the local legislation and institutional requirements. Written informed consent from the [patients/participants or patients/participants legal guardian/next of kin] was not required to participate in this study in accordance with the national legislation and the institutional requirements.

## Author contributions

XJ: Writing—original draft, Writing—review & editing, Conceptualization, Formal analysis, Methodology. YW: Investigation, Writing—review & editing, Writing—original draft.
